# Zolpidem Intoxication Associated with Probable Reverse Stress-Induced Cardiomyopathy and QT Prolongation: A Case Report

**DOI:** 10.3390/jcm15207945

**Published:** 2026-10-14

**Authors:** Hyeonbeom Byeon, Sehun Choi, Jisook Lee, Heewon Yang, Yura Ko

**Affiliations:** Department of Emergency Medicine, Ajou University School of Medicine, Suwon 16499, Republic of Korea

**Keywords:** cardiomyopathy, case report, long QT syndrome, intoxication, zolpidem

## Abstract

Zolpidem is a widely prescribed non-benzodiazepine hypnotic. A 49-year-old woman presented to the emergency department with altered mental status after intentional ingestion of approximately 300 mg of zolpidem; the blood zolpidem concentration was 674 µg/L approximately 8 h after ingestion. The initial ECG already showed QTc prolongation (499 ms). She developed chest discomfort and modest high-sensitivity troponin T elevation, and echocardiography showed transient left ventricular dysfunction (ejection fraction 42%) with basal-to-mid regional wall motion abnormalities and relative apical sparing, suggestive of probable reverse stress-induced cardiomyopathy. Coronary computed tomography angiography showed no coronary plaque or significant stenosis. On hospital day 2, telemetry captured a brief monomorphic ventricular run and QTc later peaked at 681 ms. Follow-up echocardiography on hospital day 5 showed recovery of left ventricular systolic function (LVEF 60%) and resolution of the regional wall motion abnormalities. These findings cannot be attributed solely to zolpidem. Stress may have contributed to the cardiomyopathy. Mild hypokalemia and left ventricular dysfunction may have reduced repolarization reserve. The peak QTc occurred after blood zolpidem concentrations had substantially decreased and may have reflected evolving stress-induced cardiomyopathy or concurrent exposure to cardiovascular medications. Cardiac evaluation and continuous monitoring should be considered when zolpidem intoxication is accompanied by cardiac symptoms, biomarker elevation, or ECG abnormalities.

## 1. Background

Zolpidem is a short-acting non-benzodiazepine hypnotic that selectively binds to α1 subunit-containing (ω1) gamma-aminobutyric acid type A receptors and is generally considered to have a favorable safety profile [1]. Early toxicological studies, including a retrospective review of 344 cases of acute zolpidem overdose, reported predominantly sedative toxicity with rare cardiovascular effects; cardiac biomarkers were not systematically assessed [1]. However, accumulating evidence suggests that zolpidem may exert clinically relevant electrophysiological effects on the heart [2,3,4,5].

Experimental studies have demonstrated that zolpidem acutely inhibits human ether-a-go-go-related gene (hERG) potassium channels and prolongs action potential duration in human induced pluripotent stem cell-derived cardiomyocytes, providing a mechanistic basis for acquired long QT syndrome and ventricular arrhythmias [3]. Epidemiological data further indicate that the use of non-benzodiazepine hypnotics is associated with an increased incidence of atrial fibrillation [5], and a nationwide population-based cohort study reported an increased risk of incident arrhythmia among zolpidem users [2]. A case report has described zolpidem-associated atrial fibrillation in a patient with pre-existing cardiomyopathy [4]. Despite these findings, cardiomyopathy associated with zolpidem intoxication has rarely been reported.

Stress-induced (takotsubo) cardiomyopathy is a transient, reversible left ventricular dysfunction syndrome diagnosed using accepted criteria such as the International Takotsubo (InterTAK) Diagnostic Criteria [6]; in the reverse (basal) variant, wall motion abnormalities predominantly involve the basal segments, often extending into the mid-ventricle, with relative apical sparing. In the International Takotsubo Registry, the basal type accounted for 2.2% of cases (39/1750), compared with 14.6% for the midventricular type [7]. We report a patient with confirmed zolpidem intoxication who developed a clinical phenotype consistent with probable reverse stress-induced cardiomyopathy, marked QT prolongation, and a brief ventricular arrhythmia.

## 2. Case Presentation

A 49-year-old woman presented to the emergency department (ED) with abnormal behavior. She was detained by the police after she disturbed another person’s home. When the emergency medical services arrived, she was alert but confused, lying on the floor, and gesturing aimlessly in the air. A white powder was noted in her mouth and hair, raising suspicion of drug ingestion. In the ED, the patient was alert but disoriented, without focal neurological deficits. Her initial vital signs were as follows: blood pressure, 134/83 mmHg; heart rate, 74 beats/min; respiratory rate, 18 breaths/min; body temperature, 36 °C; and oxygen saturation, 100% on room air. She had no known cardiac history and no family history of sudden cardiac death. Evaluation for possible encephalopathy and drug intoxication was initiated, including laboratory studies, electrocardiography (ECG), and brain imaging.

**Figure 1:**
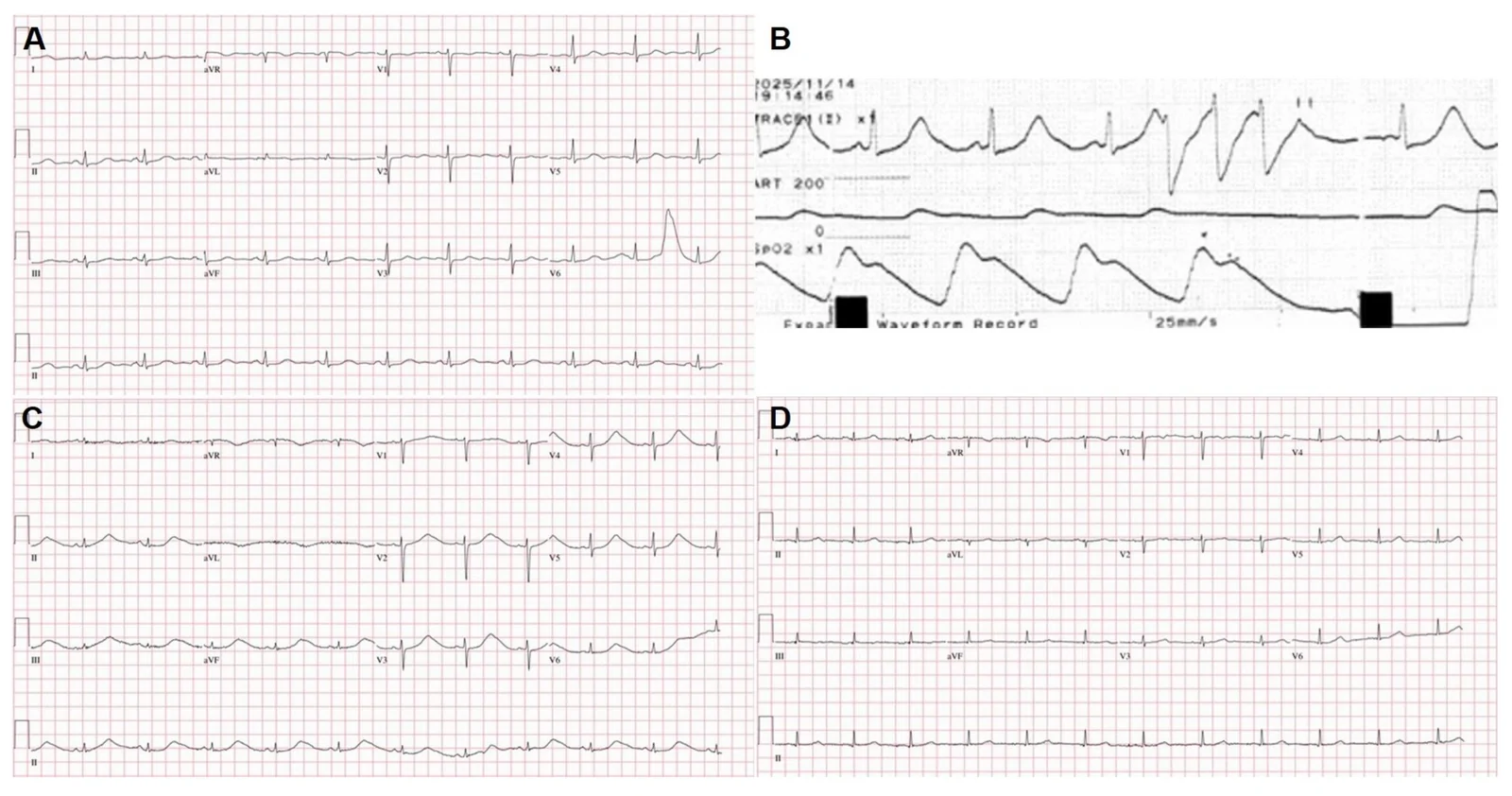
Serial ECG and telemetry findings during hospitalization. (**A**) Initial ECG showing sinus rhythm with QT/QTc intervals of 470/499 ms. (**B**) Telemetry recording (lead II) at 19:14 on HOD 2 rhythm strip showing a three-beat monomorphic ventricular run (ventricular triplet) at approximately 160–170 beats/min. Arterial pressure and SpO2 waveforms are shown below the ECG trace; paper speed, 25 mm/s. (**C**) ECG on hospital day (HOD) 2 at 19:58 showing marked QT prolongation with QT/QTc intervals of 650/681 ms. (**D**) Follow-up ECG on HOD 6 showing QT/QTc intervals of 428/465 ms. QT/QTc values in panels (**A**,**C**,**D**) were automatically generated by the 12-lead ECG system. ECG, electrocardiogram; HOD, hospital day; SpO2, peripheral oxygen saturation.

Initial laboratory testing showed a potassium level of 3.1 mmol/L, magnesium 1.9 mg/dL (reference range, 1.6–2.6 mg/dL), lactate 3.6 mmol/L, and C-reactive protein (CRP) 0.09 mg/dL; complete blood count, glucose, renal function, other electrolytes, and ammonia were within normal limits. Urine toxicology screening was negative for all nine tested categories (amphetamine, methamphetamine, barbiturates, benzodiazepines, cocaine, methadone, opiates, tetrahydrocannabinol, and tricyclic antidepressants). The serum levels of phenytoin, carbamazepine, valproic acid, digoxin, and lithium were undetectable, and the blood ethanol level was <10 mg/dL. Brain computed tomography (CT) revealed no acute intracranial abnormalities. Blood and urine specimens for comprehensive toxicological analysis were obtained at 07:54 and 08:08, respectively, approximately 8 h after the reported ingestion. Quantitative testing subsequently confirmed zolpidem concentrations of 674 µg/L in blood and 644 µg/L in urine. The blood concentration exceeded the reported therapeutic plasma range of 80–160 µg/L (up to 200 µg/L) and the reported toxic thresholds of 0.32 and 0.5 mg/L, but remained below concentrations reported in comatose or fatal cases (1.5–4 mg/L) [8]. Initial ECG showed sinus rhythm at a rate of 68 beats/min with automatically generated QT and corrected QT (QTc) intervals of 470 and 499 ms, respectively; the QTc was already prolonged at presentation (Figure 1A). Initial high-sensitivity troponin T (hs-TnT) was 45 ng/L (reference < 14 ng/L).

**Figure 2:**
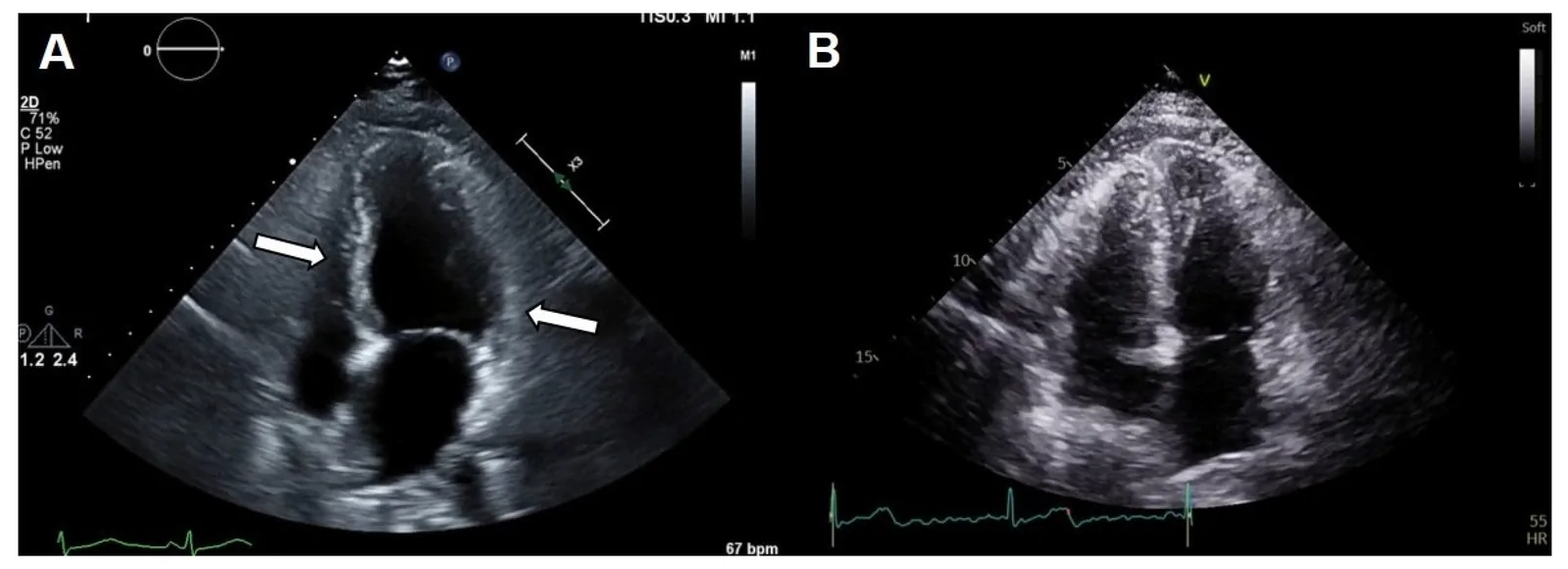
Representative end-systolic apical four-chamber TTE images. (**A**) Initial TTE showing reduced left ventricular systolic function (LVEF 42%) with regional wall motion abnormalities predominantly involving the basal-to-mid segments (arrows). (**B**) Follow-up TTE on hospital day 5 showing recovery of left ventricular systolic function (LVEF 60%) and resolution of the regional wall motion abnormalities. Dynamic findings are provided in Supplementary Videos S1 and S2. LVEF, left ventricular ejection fraction; TTE, transthoracic echocardiography.

**Figure 3:**
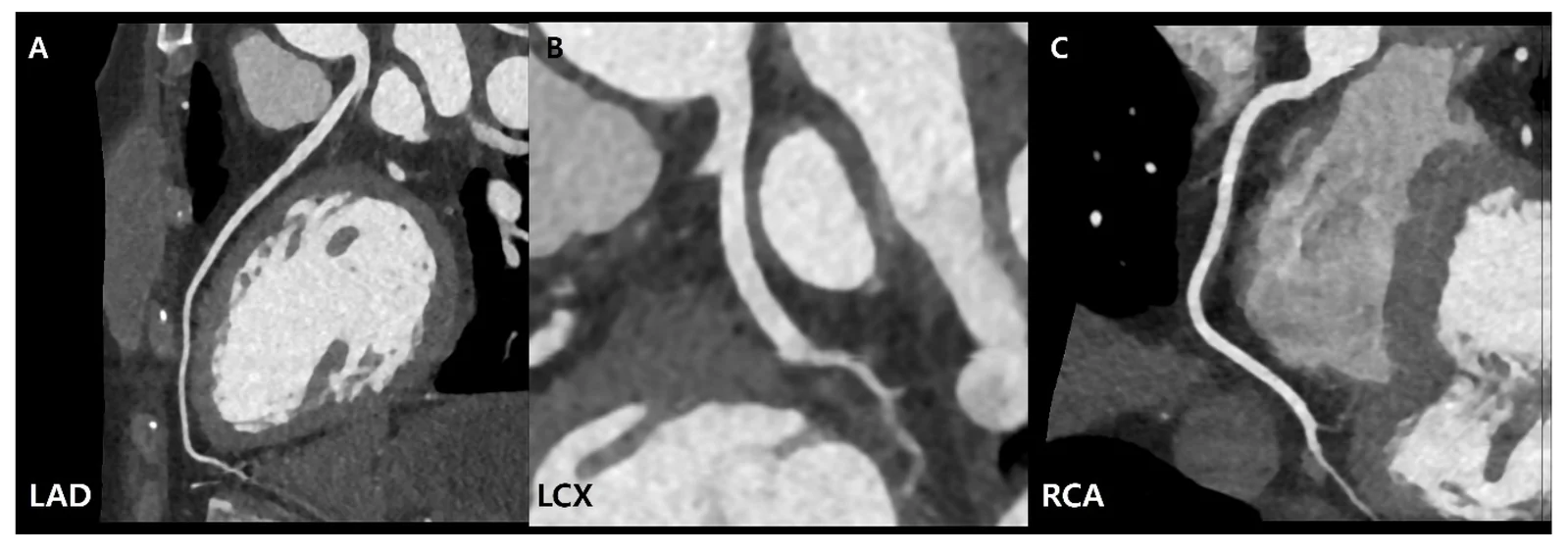
Coronary computed tomography angiography obtained on hospital day 1 demonstrating no coronary plaque or significant stenosis. (**A**) Left anterior descending artery. (**B**) Left circumflex artery. (**C**) Right coronary artery. The Agatston coronary calcium score was 0 and the examination was consistent with CAD-RADS 0; the average heart rate during acquisition was 75 beats/min.

Approximately 3 h after arrival, as her mental status improved, she disclosed that she had intentionally ingested approximately 30 tablets of her mother’s zolpidem 10 mg (approximately 300 mg) and denied any other ingestion. About 5 h after arrival, she also reported chest discomfort. Transthoracic echocardiography (TTE) showed left ventricular (LV) systolic dysfunction with an ejection fraction (LVEF) of 42% and regional wall motion abnormalities predominantly involving the basal-to-mid segments and relative apical sparing (Figure 2A, Supplementary Video S1). Repeat testing showed hs-TnT of 105 ng/L and N-terminal pro-B-type natriuretic peptide (NT-proBNP) of 528 pg/mL (reference < 249 pg/mL); total creatine kinase (CK) subsequently peaked at 756 U/L, whereas creatine kinase-MB (CK-MB) remained within the reference range (CK-MB/CK ratio < 2%). Coronary CT angiography showed no coronary plaque or significant stenosis (Agatston score 0; Coronary Artery Disease–Reporting and Data System [CAD-RADS] 0) (Figure 3). Gastrointestinal decontamination was not performed because more than 6 h had elapsed since ingestion. She was admitted to the intensive care unit for neurological and cardiac monitoring without the need for airway intervention. Serial ECG findings, cardiac biomarkers, electrolytes, and treatments during hospitalization are summarized in Table 1.

On hospital day (HOD) 2, potassium increased to 3.9 mmol/L and magnesium to 2.4 mg/dL without supplementation and the morning QTc was 483 ms. At 12:30, empagliflozin 10 mg, isosorbide mononitrate sustained-release 30 mg, and losartan 12.5 mg were each administered once. Repeat TTE showed persistent LV dysfunction (LVEF 40%). At 19:14, telemetry captured a three-beat monomorphic ventricular run at approximately 160–170 beats/min (very brief non-sustained ventricular tachycardia [NSVT]) initiated near the terminal portion of the preceding T wave (Figure 1B); the single-lead tracing was too brief to determine whether it represented torsades de pointes. A 12-lead ECG at 19:58 showed marked QT/QTc of 650/681 ms (Figure 1C). Potassium and magnesium were 3.8 mmol/L and 2.3 mg/dL, and intravenous magnesium sulfate 2 g was administered. NT-proBNP peaked at 3960 pg/mL that night.

The QTc decreased to 466 ms by the evening of HOD 3 and remained between 465 and 480 ms through HOD 6 (Figure 1D). Follow-up TTE on HOD 5 showed recovery of LVEF to 60% with resolution of the regional wall motion abnormalities (Figure 2B, Supplementary Video S2), and NT-proBNP decreased to 661 pg/mL. Holter monitoring showed sinus rhythm without recurrent arrhythmia during the recording period. Psychiatric consultation was obtained because of suicidal ideation; inpatient psychiatric care was recommended, but the patient declined and was discharged medically stable against medical advice. She returned for outpatient follow-up, although no follow-up ECG or echocardiography was performed.

This case report was prepared in accordance with the CARE guidelines. A separate patient perspective was not obtained.

## 3. Discussion

This case illustrates transient left ventricular dysfunction consistent with probable reverse stress-induced cardiomyopathy, marked QT prolongation, and a brief ventricular arrhythmia temporally associated with confirmed zolpidem intoxication. The presentation met the InterTAK Diagnostic Criteria based on the available data [6]. However, we regarded the diagnosis as probable because cardiac magnetic resonance imaging and catecholamine screening were not performed. Because the wall motion abnormalities involved both the basal and mid segments with apical sparing, the pattern was classified as the reverse (basal) type, although overlap with the midventricular type cannot be excluded. The patient had transient left ventricular dysfunction with regional wall motion abnormalities extending beyond a single coronary artery territory, electrocardiographic abnormalities, modest hs-TnT elevation, and complete recovery of ventricular function on follow-up imaging. Significant coronary artery disease does not exclude takotsubo syndrome according to the InterTAK criteria; nevertheless, coronary CT angiography showed no coronary plaque or significant stenosis in this patient. There was no clinical evidence suggesting acute infectious myocarditis: she had no fever or viral prodrome, the C-reactive protein level was normal (0.09 mg/dL), and left ventricular function and regional wall motion abnormalities recovered completely by HOD 5. In the absence of clinical red flags for acute infectious myocarditis, cardiac magnetic resonance imaging was not pursued, consistent with the diagnostic approach outlined in the InterTAK consensus document Part II [9]. Catecholamine screening was not performed; however, she had no history of paroxysmal hypertension, headache, palpitations, or diaphoresis suggestive of pheochromocytoma. Menopausal status was not documented in the available medical record; postmenopausal predominance, however, is an epidemiologic feature rather than a diagnostic requirement [6].

No specific emotional or physical trigger preceding the ingestion was identified in the available records. However, the circumstances surrounding the intentional ingestion—including acute behavioral disturbance, police detention, altered mental status, and subsequent hospitalization—may have represented physical and psychological stress and may have contributed to the development of the cardiomyopathy. Psychiatric and neurologic disorders have been reported more frequently in patients with takotsubo syndrome than in matched patients with acute coronary syndrome [7]. In this patient, psychiatric consultation was obtained because of suicidal ideation, and inpatient psychiatric care was recommended but declined. Although total CK increased approximately fourfold over 4.5 h and peaked at 756 U/L on HOD 2, CK-MB remained within the reference range (CK-MB/CK ratios, 0.38–1.91%), and the CK elevation was not interpreted as myocardial injury. NT-proBNP increased from 528 to 3960 pg/mL during persistent LV dysfunction and decreased to 661 pg/mL by HOD 5, when LVEF had recovered to 60%.

The temporal relationship between zolpidem exposure and the serial QT changes warrants careful interpretation. Assuming the longest reported elimination half-life of 5 h, the blood zolpidem concentration of 674 µg/L, measured approximately 8 h after ingestion, would have declined to about 22 µg/L by 08:33 on HOD 2, when the QTc had decreased from 509 to 483 ms, and to about 4.5 µg/L at the maximal QTc prolongation at 19:58 on HOD 2, far below the therapeutic range [8]. Persistent direct zolpidem exposure alone is therefore unlikely to explain the later increase in QTc to 681 ms. In a review of 344 cases of zolpidem overdose, electrocardiographic abnormalities were rare and none appeared directly related to zolpidem [1], and QTc prolongation was observed in only one of 74 patients in a case series of high-dose zolpidem use [10]. The measured concentration (2.19 µmol/L; molecular weight, 307.4 g/mol) was approximately 28- to 30-fold lower than the hERG IC50 values of 61.5 and 65.5 µmol/L reported by Jehle et al. [3], and the unbound concentration would be lower still because zolpidem is approximately 92% protein bound. Nevertheless, Jehle et al. observed action potential prolongation in human induced pluripotent stem cell-derived cardiomyocytes at 10 µmol/L and noted that clinically relevant proarrhythmic effects may occur in the setting of overdose or reduced repolarization reserve [3]. In this patient, the approximately 300 mg overdose, mild hypokalemia (3.1 mmol/L), and acute LV dysfunction coexisted when QTc prolongation was already evident on HOD 1 and may have increased susceptibility to the early repolarization abnormality. Although no clearly distinguishable prominent U wave was identified on visual review of the admission ECG, automated T–U discrimination could not be independently verified. However, this does not establish that direct hERG inhibition caused the later maximal QTc prolongation.

Takotsubo syndrome itself is an important competing explanation for the later repolarization abnormalities. QT prolongation and progressive T-wave inversion may occur during its evolution and provide a substrate for ventricular arrhythmias [6,9], and QTc prolongation of ≥460 ms was observed in 38% of patients in a retrospective cohort of 113 patients with takotsubo syndrome [11]. However, the ECG at maximal QTc prolongation showed diffuse, broad, low-amplitude upright T waves without T-wave inversion or a clearly separable U wave, rather than the deep, symmetric precordial T-wave inversion commonly described during the subacute phase; superimposition of a U wave could not be excluded (Figure 1C). The QT worsening also followed administration of empagliflozin, isosorbide mononitrate, and losartan; none is an established QT-prolonging drug, empagliflozin did not prolong the QT interval in a thorough QT study [12], and no related electrolyte disturbance or volume depletion was documented. These medications were therefore regarded only as potential confounders. No further doses were administered after the initial doses; however, the subsequent QT improvement cannot be attributed to this alone because intravenous magnesium sulfate was administered while the cardiomyopathy was evolving. Overall, the QT course was likely multifactorial. The early QTc prolongation was temporally associated with zolpidem exposure. However, because hs-TnT was already elevated before the first ECG, the early abnormality may also have been multifactorial. The later marked QTc prolongation occurred after zolpidem exposure had substantially declined and may have reflected evolving stress-induced cardiomyopathy, cardiovascular medication exposure, or both.

In patients with zolpidem intoxication who develop chest discomfort, cardiac biomarker elevation, or ECG abnormalities, echocardiography and continuous cardiac monitoring should be considered. Management should focus on supportive care, correction of modifiable proarrhythmic factors, and observation for evolving repolarization abnormalities and ventricular dysfunction; the optimal duration of monitoring has not been established and should be individualized.

This report is limited by its single-patient design; the absence of cardiac magnetic resonance imaging, endomyocardial biopsy, catecholamine screening, and a pre-intoxication ECG; the use of automated QT/QTc measurements without documentation of the lead, correction formula, or T–U discrimination algorithm; and the limited scope of toxicological screening, which cannot analytically exclude every QT-prolonging co-ingestant despite the patient’s denial of additional ingestion. In addition, because no pre-intoxication or outpatient follow-up ECG was available to establish her baseline QTc, an underlying predisposition to QT prolongation, including inherited long-QT syndrome, cannot be completely excluded.

## 4. Conclusions

In this patient, confirmed zolpidem intoxication was temporally associated with transient left ventricular dysfunction consistent with probable reverse stress-induced cardiomyopathy, marked QT prolongation, and a brief ventricular arrhythmia. These findings cannot be attributed solely to zolpidem: concurrent physical and psychological stress may have contributed to the cardiomyopathy, and the QT course was likely multifactorial. Cardiac evaluation and monitoring should be considered when patients with zolpidem intoxication develop chest discomfort, cardiac biomarker elevation, or ECG abnormalities.

## Figures and Tables

**Table 1:** Timeline of the clinical course, electrocardiographic findings, laboratory values, imaging, and treatment.

HOD (Date)	Time	ECG HR (bpm); QT/QTc (ms)	K (mmol/L)/ Mg (mg/dL)	Cardiac Biomarkers	Imaging	Treatment/Events
Pre- hospital	00:00 ^a^					Zolpidem ingestion, ~30 × 10 mg (~300 mg)
HOD 1	06:54					ED arrival; altered mental status
	07:54		3.1/1.9	CK 141; CK-MB 1.05; hs-TnT 45 (CK-MB/CK ratio: 0.74%)		Blood toxicology sample: zolpidem 674 µg/L
	08:08					Urine toxicology sample: zolpidem 644 µg/L
	09:57	68; 470/499				
	12:04	70; 472/509				Chest discomfort
	12:21			CK 555; CK-MB 2.87; hs-TnT 105; NT-proBNP 528 (CK-MB/CK ratio: 0.52%)		
	13:51				TTE: LVEF 42%; basal-to-mid RWMA	
	15:49				Coronary CTA: no coronary plaque or significant stenosis	
HOD 2	04:03		3.9/2.4	CK 756; CK-MB 3.55; hs-TnT 57 (CK-MB/CK ratio: 0.47%)		
	08:33	61; 480/483				
	12:30					Empagliflozin 10 mg, isosorbide mononitrate SR 30 mg, losartan 12.5 mg (single dose each)
	13:48				TTE: LVEF 40%; persistent RWMA	
	19:14					NSVT on telemetry
	19:58	66; 650/681				Maximal QTc prolongation
	20:10		3.8/2.3			
	20:17					Intravenous MgSO_4_ 2 g started
	23:17	58; 662/649				
	23:26		3.5/3.1 ᵇ	CK 348; CK-MB 1.37; hs-TnT 23; NT-proBNP 3960 (CK-MB/CK ratio: 0.39%)		
HOD 3	00:30					MgSO_4_ infusion completed
	04:09		3.6/2.7	CK 301; CK-MB 1.15; hs-TnT 20 (CK-MB/CK ratio: 0.38%)		
	08:47	57; 612/595				
	21:23	63; 456/466				QTc < 470 ms
HOD 4	04:46		3.6/2.0	CK 157; CK-MB 0.63; hs-TnT 21 (CK-MB/CK ratio: 0.40%)		
	09:44	63; 470/480				
HOD 5	04:01		3.7/1.9	CK 90; CK-MB 0.86; hs-TnT 21 (CK-MB/CK ratio: 0.96%)		
	08:20	63; 458/468				
	10:00			NT-proBNP 661		
	15:58				TTE: LVEF 60%; resolution of RWMA	
HOD 6	04:10		3.8/1.9	CK 69; CK-MB 1.32; hs-TnT 18 (CK-MB/CK ratio: 1.91%)		
	08:37	71; 428/465				Discharged in stable condition

^a^ Time of ingestion was estimated from the patient’s account. ᵇ Measured during magnesium sulfate infusion. QT/QTc values were automatically generated by the 12-lead ECG system. Reference ranges: K 3.5–5.1 mmol/L; Mg 1.6–2.6 mg/dL; CK 26–192 U/L; CK-MB < 3.61 ng/mL; hs-TnT < 14 ng/L; NT-proBNP < 249 pg/mL. Units: CK, U/L; CK-MB, ng/mL; hs-TnT, ng/L; NT-proBNP, pg/mL. bpm, beats per minute; CTA, computed tomography angiography; ECG, electrocardiography; ED, emergency department; HOD, hospital day; hs-TnT, high-sensitivity troponin T; HR, heart rate; LVEF, left ventricular ejection fraction; MgSO_4_, magnesium sulfate; NSVT, non-sustained ventricular tachycardia; QTc, corrected QT interval; RWMA, regional wall motion abnormality; TTE, transthoracic echocardiography.

## Data Availability

The de-identified clinical data supporting the findings of this case are available from the corresponding author upon reasonable request, subject to applicable ethical and privacy restrictions.

## Supplementary Materials

The following supporting information can be downloaded at https://www.mdpi.com/article/10.3390/jcm15207945/s1. Supplementary Video S1. Initial apical four-chamber transthoracic echocardiography demonstrating reduced left ventricular systolic function with regional wall motion abnormalities; Supplementary Video S2. Follow-up apical four-chamber transthoracic echocardiography on hospital day 5 demonstrating recovery of left ventricular systolic function with resolution of regional wall motion abnormalities.

## Abbreviations

CK, creatine kinase; CK-MB, creatine kinase-MB; CT, computed tomography; ECG, electrocardiography; ED, emergency department; hERG, human ether-a-go-go-related gene; HOD, hospital day; hs-TnT, high-sensitivity troponin T; LVEF, left ventricular ejection fraction; LV, left ventricular; NSVT, non-sustained ventricular tachycardia; NT-proBNP, N-terminal pro-B-type natriuretic peptide; QTc, corrected QT interval; TTE, transthoracic echocardiography.
